# Add-On Spironolactone for Persistent Proteinuria After Sodium-Glucose Cotransporter 2 Inhibitor Therapy in Patients With Diabetic Kidney Disease: A Retrospective Observational Study

**DOI:** 10.7759/cureus.102522

**Published:** 2026-01-28

**Authors:** Seigo Sugiyama, Kunio Hieshima, Noboru Kurinami, Akira Yoshida, Katsunori Jinnouchi, Tomoko Suzuki, Fumio Miyamoto, Keizo Kajiwara, Hideaki Jinnouchi

**Affiliations:** 1 Internal Medicine, Jinnouchi Hospital, Kumamoto, JPN; 2 Pharmacology, Jinnouchi Hospital, Kumamoto, JPN; 3 Ophthalmology, Jinnouchi Hospital, Kumamoto, JPN

**Keywords:** aldosterone, aldosterone antagonists, a sodium-glucose cotransporter 2 inhibitor, dkd: diabetic kidney disease, estimated glomerular filtration rate (egfr), mineralocorticoid receptor antagonist, spironolactone, type 2 diabetes

## Abstract

Background: Despite the widespread use of sodium-glucose cotransporter 2 inhibitors (SGLT2i), many patients with diabetic kidney disease continue to exhibit persistent proteinuria, highlighting an unmet clinical need for effective add-on therapies. Non-steroidal mineralocorticoid receptor antagonists (MRA) such as finerenone have recently shown benefits; however, the role of classical steroidal agents like spironolactone in this setting remains unclear.

Methods: We retrospectively analyzed 29 stable patients with type 2 diabetes mellitus (T2DM) treated at Jinnouchi Hospital who exhibited persistent proteinuria (urinary protein-to-creatinine ratio (UPCR): A2-A3; >0.15 g/g creatinine) despite more than six months of SGLT2i therapy and subsequently received add-on spironolactone for 12 months. Clinical parameters, including UPCR and estimated glomerular filtration rate (eGFR), were assessed 12 months before and at three, six, and 12 months after initiation of spironolactone therapy. We evaluated the effects of spironolactone on changes in proteinuria and the annual rate of eGFR decline.

Results: The baseline UPCR (median and interquartile range (IQR)) was 0.70 (0.40-1.27) g/g creatinine, and eGFR (mean ± standard deviation) was 49.1±16.9 mL/min/1.73 m². UPCR significantly decreased to 0.15 (0.12-0.30) g/g creatinine at 12 months after spironolactone initiation (p<0.001). Although an initial decline in eGFR was observed within three months (initial eGFR dip; -9.3%), the annual rate of eGFR decline (median (IQR)) improved from -2.2 (-7.0 to -0.9) to 1.3 (-1.5 to 3.3) mL/min/1.73 m²/year during months 3-12 after spironolactone therapy (p=0.001).

Conclusion: Add-on spironolactone therapy may provide additional renoprotective effects in patients with T2DM and persistent proteinuria despite SGLT2i treatment. Although spironolactone is an older and inexpensive steroidal MRA, it may still represent a clinically meaningful therapeutic option in the contemporary SGLT2i era.

## Introduction

Diabetic kidney disease (DKD) remains a leading cause of end-stage kidney disease (ESKD) worldwide [1]. Persistent proteinuria is a strong predictor of progressive renal dysfunction and increased cardiovascular risk in patients with DKD [2]. Large-scale clinical outcome trials have demonstrated that sodium-glucose cotransporter 2 inhibitors (SGLT2i) slow the progression of kidney disease and significantly reduce albuminuria and proteinuria [3,4]. In addition, the non-steroidal mineralocorticoid receptor antagonist (MRA) finerenone has been shown to further reduce albuminuria and attenuate the decline in estimated glomerular filtration rate (eGFR) in patients with DKD [5,6].

Accumulating evidence indicates that SGLT2i exert robust renoprotective effects, partly through reductions in intraglomerular pressure and proteinuria [3,4]. More recently, combination therapy with SGLT2i and MRAs has emerged as a promising strategy for enhanced renal protection in DKD [7]. However, a substantial proportion of patients continue to exhibit overt proteinuria despite treatment with SGLT2i alone [8]. In such patients, renal function decline often remains progressive, highlighting a persistent unmet clinical need in the contemporary SGLT2i treatment era [9].

Optimal management strategies for patients with type 2 diabetes mellitus (T2DM) who exhibit persistent proteinuria despite SGLT2i therapy remain unclear [10]. Spironolactone, a classical steroidal MRA, has long been recognized for its antiproteinuric effects [11]. Nevertheless, its clinical use has been limited by concerns regarding adverse effects, including hyperkalemia and gynecomastia [12]. Furthermore, evidence supporting the effectiveness and safety of spironolactone as an add-on therapy in the modern era, where SGLT2i are widely adopted as foundational renoprotective treatment, remains limited.

Therefore, we conducted a retrospective observational study to evaluate the real-world impact of add-on spironolactone therapy in patients with DKD who exhibited persistent proteinuria despite SGLT2i treatment. The primary endpoint was the change in urinary protein-to-creatinine ratio (UPCR) following spironolactone therapy. The secondary endpoint was the change in the annual rate of eGFR decline associated with spironolactone treatment.

This retrospective observational study aimed to evaluate the effects of add-on spironolactone therapy on residual proteinuria and renal function decline in patients with DKD who exhibited persistent proteinuria despite ongoing SGLT2i treatment.

This study was partly presented as a meeting abstract and a poster presentation at the 63rd Meeting of the Japanese Diabetes Society on May 1, 2023. The abstract was published in Japanese.

## Materials and methods

Study population and study design

This retrospective, single-center observational study enrolled adult patients with T2DM who were treated at the outpatient clinic of Jinnouchi Hospital between January 2019 and July 2022. Eligible patients had persistent proteinuria, defined as a UPCR corresponding to category A2-A3 (>0.15 g/g creatinine), despite receiving dapagliflozin (10 mg/day) for at least six months. Patients who subsequently initiated add-on spironolactone therapy at an initial dose of 12.5 mg/day were included. Participants were required to have available measurements of proteinuria and eGFR obtained both before and after the initiation of spironolactone, with a minimum follow-up duration of 12 months.

Continuation of spironolactone therapy and dose adjustments during follow-up were left to the discretion of the attending physicians, based on clinical assessments including serum potassium levels, renal function, and the degree of proteinuria reduction. Serum potassium and renal function were routinely monitored at regular outpatient visits (typically every 1-2 months) during the follow-up period, and analyses were conducted using available complete clinical data, without imputation for missing values.

Patients were excluded if they had hyperkalemia (serum potassium >5.5 mEq/L), active malignancy, severe infection, recent cardiovascular events, heart failure classified as New York Heart Association class III or higher, advanced chronic kidney disease (eGFR <15 mL/min/1.73 m²), advanced liver disease, or concomitant treatment with eplerenone.

The study was conducted in accordance with the principles of the Declaration of Helsinki. Ethical approval was obtained from the Human Ethics Review Committee of Jinnouchi Hospital (approval number: 2023-11-(2)). Written informed consent was obtained from all participants. This study was registered with the UMIN Clinical Trials Registry (UMIN ID: 000060242).

Clinical assessments and laboratory measurements

Clinical data regarding ongoing medical treatments, comorbid conditions, and medical history were collected from electronic medical records. Anthropometric and physiological parameters, including body weight, height, blood pressure, and pulse rate, were recorded during routine outpatient visits. Blood and urine samples were analyzed at the hospital laboratory. Measured laboratory parameters included plasma glucose, glycated hemoglobin A1c (HbA1c), serum creatinine, blood urea nitrogen, urinary protein, and urinary creatinine concentrations. Proteinuria was quantified using the UPCR (g/g creatinine). Semiquantitative assessment of hematuria was performed using urine dipstick testing and categorized as negative (-), borderline (±), mild (1+), moderate (2+), or severe (3+). Renal function was assessed by calculating the eGFR (mL/min/1.73 m²) using the equation proposed by the Japanese Society of Nephrology [13]. eGFR values were obtained one year prior to spironolactone initiation, at baseline, and at three, six, and 12 months after treatment initiation.

Annual changes in eGFR were calculated as follows: pre-treatment annual eGFR change was defined as the difference between the eGFR at spironolactone initiation and the eGFR measured one year earlier, while post-treatment annual eGFR change was calculated as the difference between eGFR at 12 months and at three months after spironolactone initiation, adjusted to an annualized rate.

Study outcomes

The primary outcome of this study was the change in UPCR following initiation of spironolactone therapy. The secondary outcome was the change in the annual rate of eGFR decline associated with spironolactone treatment.

Statistical analysis

Continuous variables were assessed for normality using the Shapiro-Wilk test, and variables deviating from normality were analyzed using nonparametric methods. Normally distributed variables are presented as mean ± standard deviation, whereas non-normally distributed variables are summarized as median with interquartile range (IQR). Treatment-related changes were evaluated using either the paired Student’s t-test or the Wilcoxon signed-rank test, as appropriate. Changes in eGFR over time were analyzed using repeated-measures analysis of variance (ANOVA). When the overall time effect was significant, pairwise comparisons between time points were performed using Bonferroni correction. The Greenhouse-Geisser correction was applied when the assumption of sphericity was violated. All statistical analyses were conducted using IBM SPSS Statistics for Mac, version 23 (IBM Corp., Tokyo, Japan).

## Results

Baseline characteristics

A total of 29 patients with T2DM who exhibited persistent overt proteinuria despite SGLT2 inhibitor therapy were included in the analysis. Baseline demographic, clinical, and renal characteristics are summarized in Tables 1, 2, 3.

**Table 1:** Baseline clinical characteristics Data is presented as mean+/-SD or median (IQR). CVD: cardiovascular disease; IQR: interquartile range; BMI: body mass index; PPG: postprandial plasma glucose

Characteristics	n=29
Age (years old)	68.9±10.1
Sex: male (%)	26 (89.7%)
Body weight (kg)	67.1 (62.7-79.1)
BMI (kg/m^2^)	25.0 (23.6-28.6)
Hypertension (%)	25 (86.2%)
Dyslipidemia (%)	29 (100%)
Current smoking (%)	5 (17.2%)
Diabetic retinopathy (%)	19 (65.5%)
Past history CVD (%)	15 (51.7%)
Duration of diabetes (years)	21.3±7.7
Hemoglobin A1c (%)	6.9 (5.5-7.1)
PPG (mg/dL)	148 (121-190)

**Table 2:** Baseline medical therapy ACE-i: angiotensin converting enzyme inhibitor; ARB: angiotensin-II receptor blocker; SGLT2: sodium-glucose cotransporter 2

Parameters	n=29
Glucose-lowering medications	-
Sulfonylurea (%)	6 (20.7%)
Glinide (%)	6 (20.7%)
Metformin (%)	18 (62.1%)
Alpha-glucosidase inhibitor (%)	6 (20.7%)
Thiazolidinedione (%)	1 (3.4%)
Dipeptidyl peptidase-4 inhibitor (%)	12 (41.4%)
Imeglimin (%)	0 (0%)
Glucagon like peptide-1 receptor agonist (%)	14 (48.3%)
SGLT2 inhibitor (%)	29 (100%)
Insulin (%)	12 (41.4%)
ACE-i or ARB (%)	27 (93.1%)
Statins (%)	29 (100%)
Loop diuretics (%)	2 (6.9%)

**Table 3:** Baseline renal parameters eGFR: estimated glomerular filtration rate; UPCR: urinary protein-to-creatinine ratio

Parameters	n=29
eGFR (mL/min/1.73m^2^)	49.1±16.9
Proteinuria (>30 mg/dL) (%)	29 (100%)
Occult hematuria (>10 red blood cells/mL) (%)	13 (44.8%)
Serum creatinine (mg/dL)	1.15 (0.89-1.66)
Blood urea nitrogen (mg/dL)	21.2±6.3
Serum albumin (g/dL)	4.2 (4.0-4.4)
UPCR (g/g creatinine)	0.70 (0.40-1.27)
Annual eGFR decline before spironolactone (mL/min/1.73m^2^)	-2.2 (-7.0--0.9)

Normality of continuous variables was assessed using the Shapiro-Wilk test. Several variables, including age, eGFR, systolic blood pressure, pulse rate, and serum electrolytes (Na, K, Cl), followed a normal distribution, whereas body weight, body mass index (BMI), HbA1c, postprandial plasma glucose (PPG), UPCR, and brain-natriuretic peptide (BNP) significantly deviated from normality. The corresponding W statistics and p-values are provided in the Appendix. The mean age of the cohort was 68.9 years, and 26 patients (89.7%) were male. The median BMI was 25.0 kg/m², the median HbA1c was 6.9%, and the median PPG level was 148 mg/dL. At study entry, 27 patients (93.1%) were receiving renin-angiotensin system blockade with either an angiotensin-converting enzyme inhibitor or an angiotensin II receptor blocker (Table 2). Insulin therapy was used in 12 patients (41.4%), metformin in 27 patients (62.1%), and glucagon-like peptide-1 receptor agonists in 15 patients (51.7%) (Table 2). The mean duration of diabetes was 21.3 years, and all patients were followed for 12 months. Table 3 shows the baseline renal parameters. The median baseline UPCR was 0.70 (IQR: 0.40-1.27) g/g creatinine, and the mean baseline eGFR was 49.1±16.9 mL/min/1.73 m². Occult hematuria was detected in 13 patients (44.8%) at baseline. The initial dose of spironolactone was 12.5 mg/day in all patients, and the median maintenance dose during follow-up was 25.0 (IQR: 25-25) mg/day.

Effects of add-on spironolactone therapy on proteinuria and hematuria

**Figure 1:**
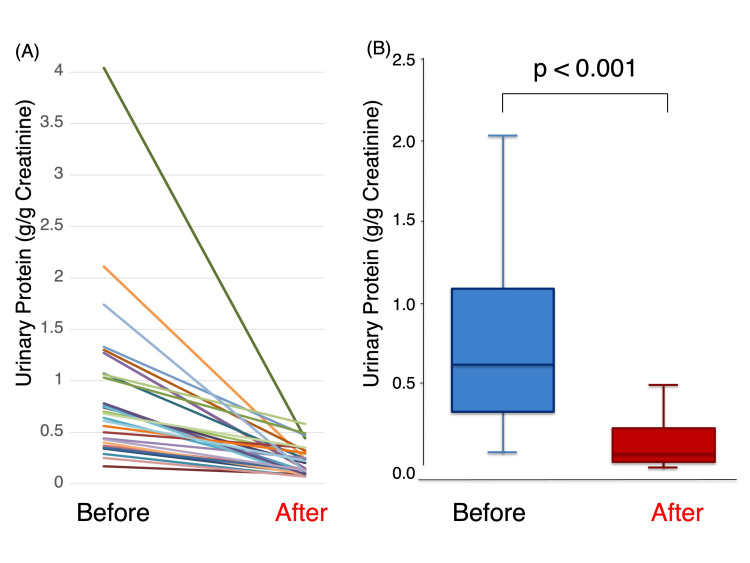
The line graph (A: individual patients) and the box-and-whisker plot (B) illustrate changes in the UPCR before and after spironolactone therapy Box-and-whisker plots (B) showing changes before and after treatment. The difference was statistically significant (Wilcoxon signed-rank test, Z=-4.703, p<0.001). UPCR: urinary protein-to-creatinine ratio

Changes in proteinuria following the initiation of spironolactone are shown in Figure 1. Median UPCR decreased significantly from 0.70 (IQR: 0.40-1.27) g/g creatinine at baseline to 0.15 (IQR: 0.12-0.30) g/g creatinine after treatment, representing a 65.5% reduction (p<0.001; Figure 1).

A reduction in UPCR was observed in all individual patients (Figure 1A). After spironolactone therapy, seven patients (24.1%) achieved remission of overt proteinuria, defined as a UPCR <0.15 g/g creatinine. Among the 13 patients with occult hematuria at baseline, an improvement in hematuria grade was observed in 11 patients (84.6%). The patterns of improvement included reductions from 3+ to 2+ (n=1), 2+ to 1+ (n=1), 2+ to ± (n=1), 1+ to negative (n=2), and ± to negative (n=6). Changes in metabolic, hemodynamic, and renal parameters during the study period are summarized in Table 4.

**Table 4:** Changes in HbA1c, s-BP, pulse rate, renal parameters, and BNP HbA1c, s-Cr, Albumin, and BNP are presented as median and IQR. s-BP, pulse rate, BUN, eGFR, Na, K, and Cl are presented as mean and +/-SD. HbA1c: glycated hemoglobin A1c; BUN: blood urea nitrogen; s-Cr: serum creatinine; eGFR: estimated glomerular filtration rate; s-BP: systolic blood pressure: Na: sodium; K: potassium; Cl: chloride; BNP; brain-natriuretic peptide; IQR: interquartile range

n=29	Before	After one year	Test statistics numerical value	P-value
HbA1c (%)	6.9 (6.6-7.1)	6.8 (6.6-7.1)	Z=-1.238	0.215
s-BP (mmHg)	126±11	120±11	t=1.874	0.071
Pulse rate (beats/min)	73±9	74±9	t=-3.829	<0.001
BUN (mg/dL)	21.2 ± 6.3	24.2±9.2	t=-2.981	0.006
s-Cr (mg/dL)	1.15 (0.89-1.60)	1.27 (0.98-1.71)	Z=-4.379	<0.001
eGFR (mL/min/1.73m^2^)	49.1±16.9	44.4±15.8	t=4.909	<0.001
Na (mEq/L)	137.4±2.4	136.9±2.7	t=1.192	0.243
K (mEq/L)	4.2±0.3	4.7±0.4	t=-4.868	<0.001
Cl (mEq/L)	102.8±2.4	103.4±3.6	t=-1.455	0.157
Albumin (g/dL)	4.2 (4.0-4.3)	4.2 (4.0-4.3)	Z=-0.943	0.347
BNP (pg/mL) n=19	54.2 (27.5-107.6)	24.6 (14.7-61.0)	Z=-3.783	<0.001

While systolic blood pressure showed a non-significant downward trend, significant reductions were observed in BNP levels and eGFR. Serum creatinine, blood urea nitrogen, and serum potassium levels increased modestly but significantly after spironolactone therapy.

Effects of add-on spironolactone therapy on eGFR

**Figure 2:**
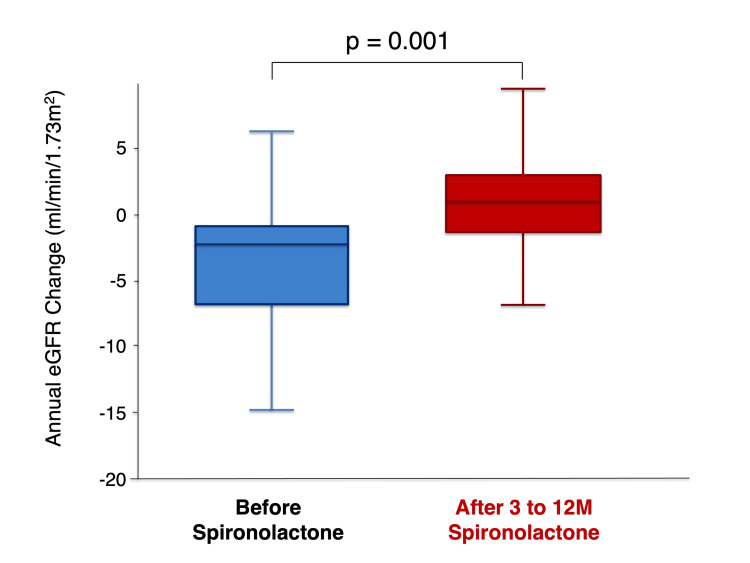
The box-and-whisker plot demonstrates changes in the annual eGFR before and after spironolactone therapy Box-and-whisker plots (B) showing changes in eGFR before and after treatment. The difference was statistically significant (Wilcoxon signed-rank test, Z=-3.541, p=0.001). eGFR: estimated glomerular filtration rate

Annual changes in eGFR before and after spironolactone therapy are presented in Figure 2.

**Figure 3:**
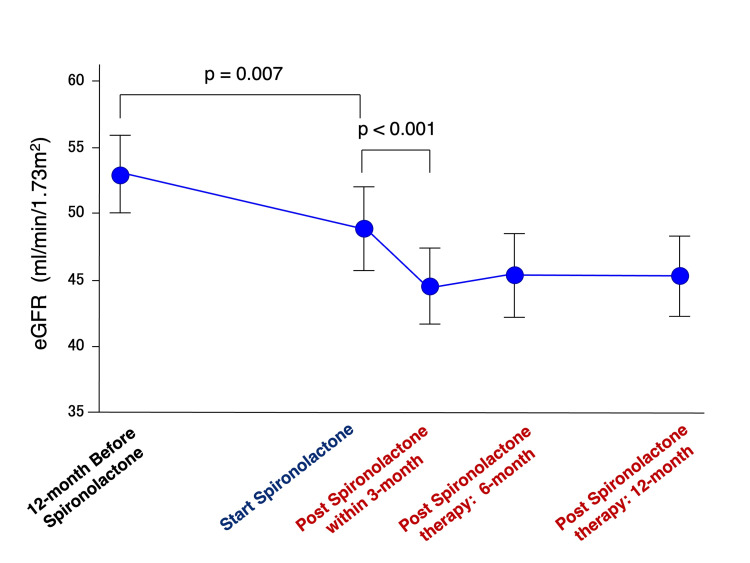
The line graph demonstrating serial changes in eGFR before and after treatment with spironolactone Data are expressed as the mean ± standard error of the mean. Repeated-measures ANOVA showed a significant overall change in eGFR across the five time points (overall p<0.05). Bonferroni-adjusted post hoc analyses indicated significant differences between one year before treatment and at treatment initiation (p=0.007), and between treatment initiation and three months (p<0.001), whereas no significant differences were observed between three months and either six or 12 months (both p=1.000). eGFR: estimated glomerular filtration rate; ANOVA: analysis of variance

The annual rate of eGFR decline improved significantly following treatment, from -2.2 (IQR: -7.0 to -0.9) mL/min/1.73 m²/year before therapy to 1.3 (IQR: -1.5 to 3.3) mL/min/1.73 m²/year after therapy (p=0.001). Serial changes in eGFR over time are illustrated in Figure 3. An initial acute decline in eGFR was observed within the first three months after spironolactone initiation (-9.3%, p<0.001), followed by stabilization during subsequent follow-up.

## Discussion

In this retrospective observational study, we demonstrated that the addition of spironolactone was associated with a marked reduction in proteinuria among patients with DKD who continued to exhibit overt proteinuria despite ongoing SGLT2 inhibitor therapy. Moreover, spironolactone treatment was accompanied by a significant attenuation of the annual rate of eGFR decline during the follow-up period. These findings suggest that spironolactone may offer incremental renoprotective benefits even in the contemporary treatment era, in which SGLT2 inhibitors constitute the foundation of guideline-based therapy [3-8].

Although both SGLT2 inhibitors and the non-steroidal MRA finerenone have been shown to slow kidney disease progression in DKD [3-7], residual proteinuria remains a strong predictor of chronic kidney disease progression and adverse renal outcomes [14]. Our results indicate that steroidal mineralocorticoid receptor (MR) blockade with spironolactone may further reduce proteinuria and stabilize renal function in patients with an incomplete antiproteinuric response to SGLT2 inhibition alone.

Spironolactone is a well-established and widely available steroidal MRA with decades of clinical experience [15]. Its efficacy in the management of hypertension and heart failure is well documented [12,16]. However, concerns regarding adverse effects, including hyperkalemia, elevations in serum creatinine, and endocrine-related side effects, have historically limited its broader application for renoprotection in DKD [12,17,18]. In the present study, spironolactone was initiated at a low dose and carefully titrated based on renal function, serum potassium levels, and clinical status. This cautious approach may be critical for maximizing antiproteinuric efficacy while minimizing treatment-related complications in patients with DKD [19,20].

From a mechanistic perspective, accumulating evidence has established a central role for aldosterone and MR activation in the progression of diabetic nephropathy [17,18]. Excess aldosterone contributes to intraglomerular hypertension through efferent arteriolar constriction and promotes glomerular capillary injury, leading to increased protein leakage [18,21]. Experimental studies have further demonstrated that aldosterone directly injures podocytes by inducing cytoskeletal disruption, reducing the expression of slit diaphragm proteins such as nephrin and podocin, and promoting podocyte apoptosis [22-24]. Consistent with these observations, MR blockade has been shown to ameliorate glomerular hyperfiltration and reduce mechanical stress on the glomerular filtration barrier [25]. In animal models of diabetic nephropathy, spironolactone preserved podocyte integrity and reduced albuminuria independently of systemic blood pressure effects, providing biological plausibility for our clinical findings [24,25].

In addition to its hemodynamic effects, aldosterone exerts pro-inflammatory and profibrotic actions within the kidney [26]. Activation of the MR enhances oxidative stress and upregulates inflammatory cytokines and profibrotic mediators, including transforming growth factor-β and connective tissue growth factor [27]. Preclinical studies have shown that spironolactone suppresses renal inflammation, reduces macrophage infiltration, and attenuates tubulointerstitial fibrosis in diabetic models [28]. These non-hemodynamic effects may be particularly relevant in DKD, where tubulointerstitial injury is a major determinant of long-term renal prognosis.

SGLT2 inhibitors primarily reduce intraglomerular pressure by restoring tubuloglomerular feedback and inducing afferent arteriolar vasoconstriction [25]. Nevertheless, residual proteinuria is frequently observed despite SGLT2 inhibitor therapy, suggesting that aldosterone-mediated pathways may continue to drive kidney injury [10]. The additive antiproteinuric effect observed with spironolactone in our cohort supports the concept of complementary mechanisms between SGLT2 inhibition and MR blockade [7]. While SGLT2 inhibitors predominantly modulate glomerular hemodynamics, spironolactone may further mitigate aldosterone-driven inflammation, fibrosis, and podocyte injury, thereby providing additional renoprotective effects.

Importantly, the reduction in proteinuria observed in this study was accompanied by a significant improvement in the annual rate of eGFR decline after spironolactone initiation. Given the well-established association between residual proteinuria and subsequent renal outcomes, these findings raise the possibility that spironolactone-induced proteinuria reduction may translate into long-term renal benefit in selected patients [14].

Although recent large-scale trials have highlighted the efficacy of non-steroidal MRAs such as finerenone, spironolactone remains a cost-effective and widely accessible therapeutic option in routine clinical practice. Our findings suggest that even low-dose spironolactone may confer clinically meaningful benefits in patients with persistent proteinuria after SGLT2 inhibitor therapy, provided that careful patient selection and close monitoring for adverse events are ensured.

Clinical implications

Spironolactone may represent a practical and cost-effective add-on therapeutic option for patients with DKD who continue to exhibit proteinuria despite guideline-recommended SGLT2 inhibitor therapy.

Limitations

This study has several limitations that warrant consideration. First, the retrospective and single-center design, along with the relatively small sample size, limits the ability to draw definitive causal inferences and introduces the potential for selection bias. Second, spironolactone dosing was individualized according to clinical judgment, which may have contributed to variability in treatment response. Third, the absence of a comparator group precludes direct attribution of observed changes solely to spironolactone therapy. Fourth, the potential confounding from concomitant medications or lifestyle factors was not fully controlled. Finally, as an exploratory analysis conducted in clinically stable patients receiving contemporary background therapy, including renin-angiotensin system inhibitors, SGLT2 inhibitors, and statins, the generalizability of our findings to broader DKD populations may be limited. Prospective controlled studies are needed to confirm these observations.

## Conclusions

Add-on spironolactone therapy was associated with significant reductions in proteinuria and attenuation of the annual eGFR decline in patients with T2DM and persistent proteinuria despite SGLT2 inhibitor therapy. Although spironolactone is an older and inexpensive agent, it may still play a meaningful role in the current clinical management of DKD when used judiciously.

## Appendices

**Table 5:** Normality assessment of continuous variables eGFR: estimated glomerular filtration rate, BNP: brain-natriuretic peptide; BMI: body mass index; UPCR: urinary protein-to-creatinine ratio; PPG: postprandial plasma glucose

Variable	Shapiro-Wilk W	P-value	Normality (p≥0.05)
Age	0.987	0.776	Yes
Body weight	0.789	<0.001	No
BMI	0.878	0.003	No
Duration of diabetes	0.933	0.067	Yes
Hemoglobin A1c	0.668	<0.001	No
PPG	0.925	0.04	No
eGFR	0.979	0.819	Yes
Serum creatinine	0.896	0.008	No
Blood urea nitrogen	0.982	0.878	Yes
Serum albumin	0.820	<0.001	No
UPCR	0.727	<0.001	No
Annual eGFR decline before spironolactone	0.894	0.007	No
Systolic blood pressure	0.981	0.866	Yes
Pulse rate	0.968	0.508	Yes
Sodium (Na)	0.958	0.287	Yes
Potassium (K)	0.940	0.103	Yes
Chloride (Cl)	0.970	0.565	Yes
BNP	0.569	<0.001	No
